# Damage to the blood-aqueous barrier in eyes with primary angle closure glaucoma

**Published:** 2010-10-08

**Authors:** Xiangyun Kong, Xing Liu, Xiangkun Huang, Zhen Mao, Yimin Zhong, Wei Chi

**Affiliations:** State Key Laboratory of Ophthalmology, Zhongshan Ophthalmic Center, Sun Yat-Sen University, Guangzhou, People’s Republic of China

## Abstract

**Purpose:**

This study investigates the inflammation in the anterior chamber in eyes with primary angle closure glaucoma (PACG) and evaluates the effect of intraocular pressure (IOP) elevation on the blood-aqueous barrier (BAB).

**Methods:**

Thirty-five patients (35 eyes) with acute primary angle closure glaucoma (APACG), 42 patients (42 eyes) with chronic primary angle closure glaucoma (CPACG), and 50 age-matched healthy controls (50 eyes) were included in this study. The flare value and cell counts were quantified using laser flare cell photometry. Statistical analysis was performed to compare differences in flare value and cell counts between different groups and explore the relation between the inflammation and IOP.

**Results:**

The mean flare value (photon counts per millisecond, ph/ms) in the APACG, CPACG, and healthy control group was 141.4±123.1, 7.7±4.1, and 4.5±1.1, respectively. The mean cell counts (cells/0.5 mm^3^) in the three groups were 126.0±67.8, 5.2±5.8, and 0.8±0.7, respectively. The flare value and cell counts in both the APACG group and the CPACG group were significantly higher than those in the healthy control group (p<0.001). Furthermore, the flare value and cell counts in the APACG group were significantly higher than those in the CPACG group (p<0.001). There were positive correlations between the IOP level and flare value (r=0.527, p<0.001), and cell counts(r=0.775, p<0.001), respectively, in the APACG group.

**Conclusions:**

Disrupted BAB and inflammation in the anterior chamber were found in eyes with both kinds of PACG. The damage of BAB was more severe in eyes with APACG than those with CPACG. The IOP elevation, especially a dramatic IOP elevation, might be the factor responsible for the change of BAB in eyes with PACG.

## Introduction

Primary angle closure glaucoma (PACG) is the most common type of glaucoma in the Asian population, and it is responsible for most of bilateral glaucoma blindness in China [1]. As a common complex disease, it has become an important target for association studies in recent years [2]. Several previous studies focusing on the pathogenesis of PACG have suggested that the geometry of the anterior chamber [3], certain genes [4-7], and several other risk factors [8] are related with PACG. Yet, the pathological change in PACG has not been clearly investigated. The alteration of the blood-aqueous barrier (BAB), which is located in the apico-lateral surfaces of the non-pigmented epithelium of the ciliary body and between the endothelial cells of the iris vasculature [9], has been unknown. The BAB shows a high degree of selectivity that prevents the passage of plasma proteins into the aqueous humor, which helps to keep a very low protein concentration and rare cells of aqueous humor to maintain optical clarity and prevent light scattering. It was reported that the control of the barrier function is influenced by the flow of aqueous humor in the routes between Schlemm's canal and the anterior chamber [10]. Since PACG always has an elevation of intraocular pressure (IOP) resulting from the abnormality in the aqueous humor dynamics, it seems that the BAB may be affected in eyes with PACG.

Aqueous flare and cells are generally considered to be two inflammatory parameters of anterior chamber inflammation resulting from destruction of the BAB. Despite various characteristic clinical signs such as conjunctival congestion, corneal epithelial edema, middilated unreactive pupil, glaucomflecken, and iris atrophy, tyndallometry is frequently observed in eyes with acute primary angle closure glaucoma (APACG) soon after an attack episode during the slit-lamp biomicroscopy examination. Because the slit-lamp biomicroscopy examination is qualitative and subjective with considerable intra- and inter-observer variations, there has been no quantitative data referring to the anterior chamber inflammation in eyes with APACG. On the other hand, the aqueous flare and cells are rarely seen in eyes with chronic primary angle closure glaucoma (CPACG) in the slit-lamp biomicroscopy examination. Previously, there has been no published data on the change of the BAB in eyes with CPACG.

Laser flare cell photometry (LFCM) represents the first noninvasive, objective, and quantitative method to assess intraocular inflammation [11]. It also allows the accurate detection of subclinical alterations in the BAB [11]. With the application of laser flare cell photometry, the changes of the BAB have been studied in a variety of ocular diseases such as uveitis [12-14] and noninflammatory diseases [15-17]. Laser flare cell photometry also shows utility in the comparison of the effects on the BAB of different surgical techniques [18,19], surgical adjuncts [20], laser procedures [21,22], and several new medical managements of glaucoma [23,24]. However, laser flare photometry has not yet been applied to study the change of the BAB in eyes with PACG. Neither has the effect of IOP elevation on the permeability of the BAB been investigated.

In this study, we used laser flare cell photometry for aqueous flare and cells measurements to both quantitatively evaluate the inflammation in the anterior chamber in eyes with PACG and explore the influence of IOP elevation on the BAB.

## Methods

The study was conducted at Zhongshan Ophthalmic center of Sun Yat-sen University, Guangzhou, People’s Republic of China from 1 October 2008 to 30 September 2009 with the approval of the Ethics Committees of the Zhongshan Ophthalmic Center and in accordance with the tenets of the Declaration of Helsinki. All patients and healthy volunteers were informed about the nature of the noninvasive examinations of LFCM. Informed written consents were obtained.

The study comprised three groups of participants: the APACG group, the CPACG group, and the age-matched healthy control group. The diagnostic criteria of the APACG group were the following: 1) the presence of at least two of the following symptoms: eye pain, headache, blurred vision, and vomiting; 2) the presence of at least three of the following signs: conjunctival congestion, corneal epithelial edema, middilated unreactive pupil, glaucomflecken, and iris atrophy; 3) 270 degree or greater of anterior chamber angle closure on gonioscopic examination; and 4) IOP>40 mmHg by Glodmann applanation tonometry.

The diagnostic criteria of the CPACG group were the following: 1) the presence of glaucomatous optic neuropathy, which was defined as a cup-to-disc ratio ≥0.7 or asymmetry ≥0.2 between the two eyes, neuroretinal rim width reduced to ≤0.1 cup-to-disc ratio, and nerve fiber layer defect; 2) visual field loss detected with static automated white-on-white threshold perimetry (SITA Fast strategy, program 30–2, model 750; Humphrey Field Analyzer; Carl Zeiss Meditec, Dublin, CA) that is consistent with glaucomatous optic nerve damage. This was established on Glaucoma Hemifield test results outside normal limits and/or an abnormal pattern standard deviation (SD) with p<5% occurrence in the normal population; and 3) a closed angle on indentation gonioscopy, which was defined as the presence of at least a 180° angle in which the posterior pigmented trabecular meshwork was not visible and with evidence of peripheral anterior synechiae in any part of the angle. These criteria were in compliance with the International Society for Geographical and Epidemiological Ophthalmology (ISGEO) classification of angle-closure glaucoma by Foster et al. [25].

In eyes with APACG that had a recent attack within 7 days, the IOP was controlled and the cornea without edema were eligible for the participation of the APACG group, while eyes that were first diagnosed with CPACG and had not received any systemic or topical treatment to reduce the IOP were eligible for the CPACG group. The time interval between the acute attack and the laser flare cell photometry examination in the APACG eyes was 89.5±47.5 h (range 11~168 h). Eyes were excluded if they had secondary angle closure or conditions that might influence the accuracy of measurement or associate with impairment of the BAB such as ocular inflammatory disease, overt cataract, retinal vein occlusion, retinal detachment, or a personal history of hypertension, diabetes, rheumatism, or previous intraocular surgical or laser procedures.

All the control subjects received a complete ocular examination, which included best corrected visual acuity measurements using the logarithm of the minimum angle of resolution 4-m charts, slit-lamp evaluation of the anterior segment, fundus examination, measurement of IOP, axial length measurement (A-scan ultrasonography; Quantel Medical, Clermont-Ferrand, France), and detailed recording of the health and degree of cupping of the optic nerve head. The inclusion criteria of the control group were the following: 1) age-matched to the APACG group and CPACG group ; 2) had open anterior chamber angles; 3) had normal optic nerve heads with cup-to-disc ratio of ≤0.5; 4) IOPs <21 mmHg ; 5) did not have a history of any of the aforementioned symptoms and signs; 6) had no ophthalmic diseases except mild cataract; 7) had no personal history of hypertension, diabetes, or rheumatism; and 8) had no previous intraocular surgical or laser procedures. To reduce an undue contribution from a single patient, if both eyes were eligible for the study participation, only the right eye served.

The quantitative measurement of aqueous flare and cells was performed by an experienced technician, who did not know the clinical results, with the use of laser flare cell photometry (FC-2000; Kowa Co., Ltd., Tokyo, Japan). The amount of light scattered by solutes (protein) or particles (cells) in the anterior chamber was detected by photomultipliers in the machines. For measurement of protein concentration (flare), the devices recorded the amount of light detected by the photomultiplier as it was scanned across a window measuring 0.3 mm×0.5 mm over 0.5 s, and it recorded two background readings that were taken as the laser scans above and below the window. The background measurements were averaged and subtracted from the reading obtained in the scanned window to provide a laser flare photometry measurement. For the cell count, two optical scanners were operated in synchronization to scan a fixed volume (0.5 mm^3^) two-dimensionally. When the laser light passed across a large particle (a cell) a strong peak was produced. The number of peaks in the fixed volume was counted to give the number of cells [11]. To ensure the accuracy of the measurements, calibration was performed each day according to the instructions of the company. For each eye, seven individual measurements were obtained in which the difference between background values was 15% or less. The highest and lowest values were discarded and the remaining five values were averaged [26]. Flare and cell readings were expressed as photon counts per millisecond (ph/ms) and cells/0.5 mm^3^, respectively.

The data were processed and statistically analyzed using SPSS for Windows (Version 13.0; SPSS, Chicago, IL). All the results of age, IOP, and laser flare cell photometry were checked for normal distribution. A χ^2^ test was used to compare the male/female ratio. For comparison between the two different groups, a two-sample Student *t*-test was used to evaluate differences in average between normal distributed data while the Mann–Whitney U-test was used when abnormal distributed data existed. One-Way ANOVA was used to compare the average among normal distributed data in the three groups while the Kruskal–Wallis H test was used when abnormal distributed data existed. Spearman correlations analysis was used for bivariate correlations analysis between flare value and IOP levels, cell counts, and IOP levels in the APACG and the CPACG groups. A p<0.05 was considered statistically significant.

## Results

### Subjects in the APACG, CPACG, and healthy control groups

In total, there were 35 eyes of 35 individuals in the APACG group, 42 eyes of 42 individuals in the CPACG group, and 50 eyes of 50 individuals in the healthy control group, respectively. The cases, male to female ratio, average age, and mean IOP level of each group are summarized in Table 1.

**Table 1:** The participants’ characteristics.

**Group**	**APACG**	**CPACG**	**Healthy control**	**p**
Number of eyes	35	42	50	–
Gender (male/female)	14/21	20/22	27/27	0.650*
Age	60.7±10.1	62.2±8.8	62.5±11.0	0.714**
IOP (mmHg)	57.0±10.6	35.5±10.2	14.2±2.5	<0.001#

The asterisk indicates the p value of the comparison among the three groups was determined by the χ^2^ test. The double asterisk indicates that the p value of the comparison among the three groups was determined by One-Way ANOVA. The sharp (hash mark) indicates that the p value of the comparison among the three groups was determined by the Kruskal–Wallis H test. Abbreviations: APACG (acute primary angle closure glaucoma), CPACG (chronic primary angle closure glaucoma), IOP (intraocular pressure).

Comparison among the three groups did not reveal significant differences in both the male/female ratio (p=0.650) and mean age (p=0.714). The attack episode IOP level in the APACG group, the first visit IOP level in the CPACG group and the examination IOP level in the healthy control group were 57.0±10.6 mmHg (range 42~94.3 mmHg), 35.5±10.2 mmHg (range 22~62 mmHg), and 14.2±2.5 mmHg (range 8~19 mmHg), respectively. There was a significant difference in the mean IOP level among the three groups (p<0.001). The APACG group had significantly higher IOP level than CPACG group (p<0.001), while the CPACG group had a significantly higher IOP level than the healthy control group (p<0.001).

### The anterior chamber flare value in the APACG, CPACG, and healthy control groups

The anterior chamber flare value measured by laser flare cell photometry in the APACG group, CPACG group, and healthy control group is showed in Table 2. The flare value was abnormally distributed in all except healthy control group. The Kruskal–Wallis H test showed that the difference in the flare values among the three groups was significant (p<0.001). Both the APACG group and the CPACG group had significantly higher aqueous flare value in the anterior chamber than that of healthy control group (p<0.001). Furthermore, the flare value in the anterior chamber in the APACG group was significantly higher than that in the CPACG group’s eyes according to the Mann–Whitney U test (p<0.001).

**Table 2:** Comparison of flare values (ph/ms) among the APACG, CPACG, and healthy control groups.

**Group**	**APACG (n=35)**	**CPACG (n=42)**	**Healthy control (n=50)**
mean±SD	141.4±123.1	7.7±4.1	4.5±1.1
Median (interquartile range)	75.2(238.8)	6.7(4.1)	4.4(1.6)
Range	13.5~371.3	3.2~26.7	2.7~7.4
p*	<0.001	<0.001	–

The asterisk indicates the p value compared with the healthy control group. Abbreviations: ph/ms (photon counts per millisecond), APACG (acute primary angle closure glaucoma), CPACG (chronic primary angle closure glaucoma).

### The anterior chamber cell counts in the APACG, CPACG, and healthy control groups

The anterior chamber cell counts measured by laser flare photometry in the APACG group, CPACG group, and healthy control groups are showed in Table 3. The cell counts in the APACG group were all abnormally distributed. The cell counts of 3 eyes in the APACG group could not be obtained. Significant differences in the cell counts among the three groups were showed by the Kruskal–Wallis H test (p<0.001). The cell counts either in the APACG group or in the CPACG group were significantly higher than those in the healthy control group. Moreover, the cell counts in the APACG group were significantly higher than those in the CPACG group (p<0.001).

**Table 3:** Comparison of the cell counts (cells/0.5 mm^3^) among the APACG, CPACG and healthy control groups.

**Group**	**APACG (n=32)**	**CPACG (n=42)**	**Healthy control (n=50)**
mean±SD	126.0±67.8	5.2±5.8	0.8±0.7
Median (interquartile range)	111.4 (80.7)	2.8 (6.7)	1 (1.4)
Range	21.5~312.2	0~27.3	0~2.7
p*	<0.001	<0.001	–

The asterisk indicates the p value compared with the healthy control group. Abbreviations: APACG (acute primary angle closure glaucoma), CPACG (chronic primary angle closure glaucoma).

### The correlation of the IOP and flare value and the IOP and cell counts in eyes with APACG and CPACG

**Figure 1:**
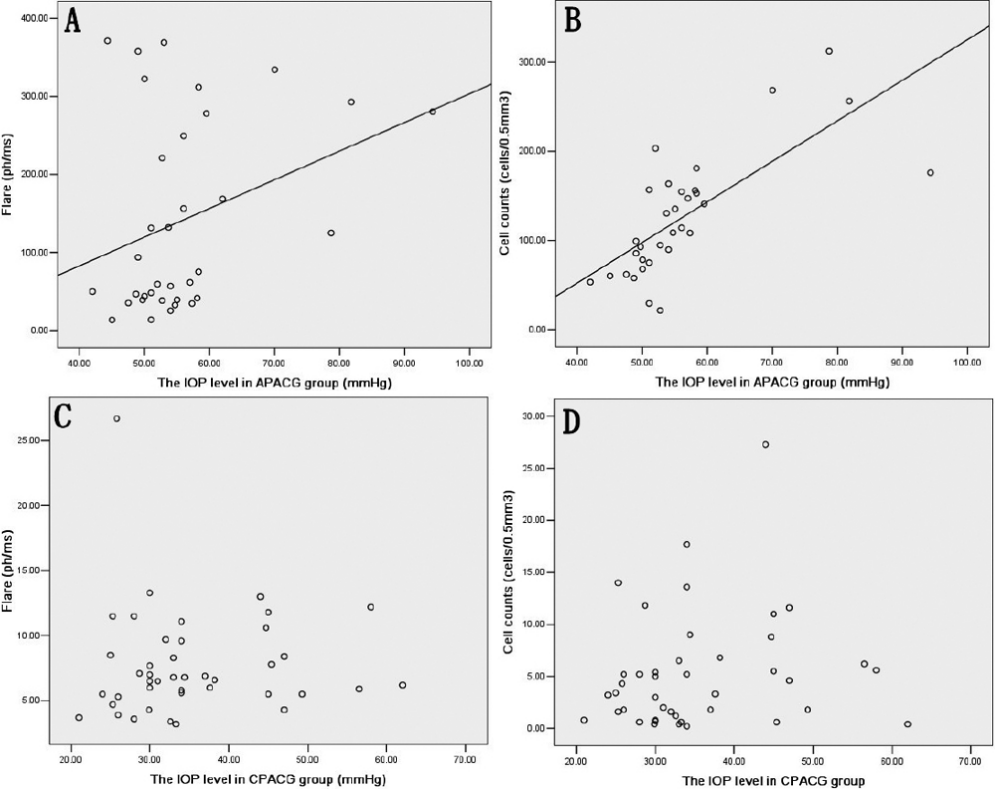
The relation between the IOP and flare and the IOP and cell in the APACG and CPACG group. **A**: The flare was positively related with the intraocular pressure (IOP) in the acute primary angle closure glaucoma (APACG) group (r=0.527, p<0.001). **B**: The cell was positively related with the IOP in the APACG group (r=0.775, p<0.001). **C**: There was no significant correlation between the IOP and flare in the chronic primary angle closure glaucoma (CPACG) group (r=0.131, p=0.408). **D**: There was no significant correlation between the IOP and cell in the CPACG group (r=0.158, p=0.336).

The Spearman correlation coefficient between the IOP and flare value was 0.527 (p<0.001) and between IOP and cell counts was 0.775 (p<0.001) in eyes with APACG, while it was 0.131(p=0.408) and 0.158 (p=0.336) in the CPACG group, respectively (Figure 1).

## Discussion

The effective BAB keeps the aqueous humor almost completely protein and cell free. Thus, the emergence of aqueous flare and cells indicates anterior chamber inflammation due to the impairment of the BAB. Artificial grading of aqueous flare and cells observed by slit-lamp biomicroscopy is traditionally used to evaluate the severity of BAB disruption and anterior chamber inflammation [27]. However, when examined with the slit lamp, the measurement of intraocular inflammation remains subjective with considerable intra- and inter-observer variations. With the use of LFCM, we found that the flare value and cell counts in the APACG group and the CPACG group were significantly higher than those in the age-matched healthy control group (p<0.001). These results suggested that the BAB was impaired and the inflammation was presented in the anterior chamber of eyes with two kinds of PACG. LFCM demonstrated a particular advantage in evaluating the permeability of the BAB and the inflammation in the anterior chamber both objectively and quantitatively in eyes with APACG. It also allowed the detection of subclinical alterations to observe subtle pathological changes in the BAB of eyes with CPACG.

It was reported that the BAB can be broken down by a variety of agents such as prostaglandin and non-prostaglandin [28]. In addition, the permeability of the BAB shows a significant degree of pressure-dependent diffusion associated with transport activity, resembling the standing gradient osmotic flow model [29]. The effect of osmotic shock on the BAB was investigated as early as in the 1970s [30]. However, the effect of IOP elevation as a potential mechanical shock on the BAB has not been studied until the present.

Because age was reported as perhaps relating to BAB instability [31], and several medical managements of glaucoma were proved to be able to affect the permeability of BAB [23,32], eyes with APACG that had a recent attack within 7 days and eyes which were first diagnosed with CPACG without using any systemic or topical treatment to reduce IOP were enrolled in the PACG group in this study. The effect of IOP elevation on the BAB was accessed by comparing flare value and cell counts of the two PACG groups to those of the age-matched healthy control group. Our study showed that both the APACG group and the CPACG group had significantly higher IOP levels and corresponding significantly higher flare value and cell counts than the healthy control group, implying that IOP elevation might be responsible for the changes of the BAB in the two PACG groups.

Compared with the CPACG group, the inflammation in the anterior chamber in the APACG group was more severe. Correspondingly, the IOP level in the APACG group was significantly higher than in the CPACG group (p<0.001). The IOP elevation was sudden once the APACG attacked. The speed of IOP elevation in eyes with APACG was much more acute than in eyes with CPACG. Furthermore, the anterior segment ischemia (ASI) always occurred when the IOP elevated suddenly [33]. Aung and his associates [33] examined the iris ischemic change (IIC)—the sign of ASI—in 61 subjects with episode of acute primary angle closure. The results showed that more than half the subjects (52.5%) were found to have developed IIC during the study, 65% of whom already had signs of IIC by the first week. The ASI caused the protein influx into the anterior chamber, which aggravated the disruption of the BAB [34]. The great mechanical pressure of sudden markedly elevated IOP and the presence of ASI in the eyes with APACG led to the inflammation in the anterior chamber. Our results showed that there were positive correlations between IOP and flare value and cell counts in eyes with APACG, which meant that the higher the IOP elevation, the greater the mechanical pressure, the more susceptible the ASI, and the more severe the inflammation presented in eyes with APACG. However, in eyes with CPACG, the IOP elevation was latent and mild, and IIC was rarely found. Furthermore, the IOP level did not relate to the mechanical pressure of the BAB directly because of the slow speed of IOP elevation. Thus, the breakdown of the BAB in eyes with CPACG was not as severe as that in eyes with APACG. The IOP level did not associate with the flare value and cell counts.

The possibility that IOP could be affected by the increased permeability of the BAB has been reported [35,36]. In addition, the elevated protein content, inflammatory cells and the mediators, including cytokines and chemokines, are considered to play an important role in the pathogenesis of secondary elevated intraocular pressure (IOP) and uveitic glaucoma [37,38]. However, because the systemic or topical treatments were immediately used to reduce the IOP once APACG was diagnosed in our study, we could not observe any effect of increased permeability of the BAB on the IOP.

Surgical procedure is the main treatment to break the pupillary block and control the IOP for eyes with PACG. It is reported that filtering surgery in severely inflamed eyes is likely to lead to a high failure rate and a high rate of complication incidence [39,40]. Therefore, in addition to controlling IOP, anti-inflammation treatments are necessary for eyes with PACG, especially APACG,which can be helpful in both improving the recovery of the BAB and increasing the success rate of anti-glaucoma surgery.

In conclusion, our results demonstrated that the BAB was impaired and inflammation was present in the anterior chamber in eyes with APACG and CPACG. IOP elevation is a possible factor that causes the impairment of the BAB in APACG. Furthermore, the ASI due to the sharp IOP elevation may be the other risk factor that aggravates the destruction of the BAB in APACG. The exact mechanism of the effect of IOP and ASI on the BAB remains unclear. Attention should be drawn to changes in the BAB in eyes with PACG.
